# Application and enlightenment of mobile hospital in medical support for sports event

**DOI:** 10.3389/fpubh.2025.1642255

**Published:** 2025-07-28

**Authors:** Bo Jia, Jian Zhang, Ang Zhang, Jiaqi Zhu, Mengyun Sui, Long Xue

**Affiliations:** ^1^Huashan Hospital, Fudan University, Shanghai, China; ^2^Shanghai Municipal Center for Disease Control & Prevention, Shanghai, China

**Keywords:** mobile hospital, sports event, medical support, emergency medical service, disaster medicine

## Abstract

With the vigorous development of sports events, the rising medical demand during the events has brought a heavy burden to urban emergency medical services. On the other hand, mobile hospitals, featuring strong mobility, high environmental adaptability, rapid deployment, and comprehensive functions, have shown great application value. This study provides an analysis of the unique advantages of mobile hospitals in rapid response and deployment, provision of on-site comprehensive medical services. Through literature review of the application of mobile hospital, the observation of mobile hospitals in the 2024 Tour Shanghai New City Cycling Race and the 2024 Shanghai Marathon, it is found that in sports events involving complex races such as cross-administrative regions and multi-stage races, mobile hospitals have significant application advantages, especially in improving the overall level of medical security of the race and significantly reducing the workload of emergency medical treatment. It shows great development potential.

## Introduction

In recent years, the scale and influence of major sporting events have continuously growth, drawing tens of thousands of participants and spectators (1, 2). Such events are often located in urban core areas or span multiple administrative jurisdictions, involving vast geographical areas and dense crowd gatherings, which presents significant challenges for medical support (3). Against this backdrop, establishing comprehensive medical emergency preparedness to ensure participants and spectators receive timely medical services is crucial (4).

Meanwhile, emergency medical (EM) systems worldwide are experiencing increasingly overcrowding. This phenomenon primarily stems from two factors: on one hand, the continuous growth of healthcare demand; on the other hand, the disproportionate utilisation of EM by an ageing population due to chronic illnesses (5, 6). Mass gathering often have unpredictable impacts on the local EM systems. The uncertainty of potential incidents during events, combined with the possibility of widespread EM needs, highlight the imperative for EM preparedness (1).

As a flexible and efficient on-site medical solution, mobile hospitals (MH) have shown advantages in various EM scenarios, including disaster relief (7). It has three characteristics: rapid deployment capability, a modular design approach, and comprehensive EM service functions (8), make it ideal for ensuring medical safety at major sporting events.

Through a literature review and observations of practical of MH applications in sport events, This study aims to systematically explore the value of MH in providing EM support within complex event environments, particularly large-scale sporting events spanning multiple administrative regions and stages.

## Definition and functions of MH

MH are modularly designed and adaptable medical systems. They facilitate early medical intervention and specialised EM services through the flexible combination of standardised functional units, typically comprising medical, technical, ward, and life support components (9). These units have strong mobility, high environmental adaptability, rapid deployment capabilities, and integrated functionalities (8). This allows them to deliver near-hospital level EM services directly when need.

MH has played an important role in the rescue of major disasters such as the Haiti earthquake (10), the Philippines typhoon (11), Nepal earthquake (12), and the Wenchuan earthquake (13) and Lushan earthquake (14) in China. The core value of MH lies in their capacity to manage high-risk cases on the spot, thereby significantly reducing the burden on EM departments, avoiding unnecessary patient referrals, and optimising the efficiency of EM resource allocation. As a result, they are increasingly recognised as an ideal solution for addressing the complex and diverse medical support requirements of sporting events.

## Advantages of MH in medical supports for sports events

MH establish a full-chain EM service of “on-site first aid, precise diagnosis and treatment, and continuous care.” This ensures dynamic alignment between medical resources and event demands. Their advantages are reflected in four dimensions:

### Advancement of EM and improvement of diagnosis and treatment efficiency

MH integrate surgical, X-ray, and laboratory equipment, enabling it to provide hospital-equivalent EM capabilities directly at on-site (15). This includes the management of event-related acute conditions such as shock resuscitation, cardiac arrest, debridement and suturing, and fracture immobilisation. MH’s modular diagnostic units, comprising X-ray, B-mode ultrasound, and laboratory testing systems, greatly reduce on-site diagnostic times. This forward-deployed capability allows for immediate management of injuries and illnesses at the event, thereby minimising unnecessary referrals and effectively alleviating pressure on EM departments.

### Medical cost control and medical resources optimization

International practices demonstrated that the medical model of MH is cost-effective. Examples include Hospital in the Nursing Home (HiNH) in Australia (6), HAH (Hospital at Home) in France (16), and Pathfinder in Ireland (17), which have consistently shown that integrating MH services into aged care facilities can substantially reduce EM visit and hospital admissions. For example, the HiNH programme savde a AU$8,659,788 per year by reducing EM utilisation, substantially exceeding its operational costs of AU$488,116, resulting in a net annual saving of AU$8,171,671 (18). The triage mechanisms and demand management strategies employed by these programmes are directly applicable to event medical support, reducing reliance on visits and hospitalizations of EM.

### Improving resilience of city

MH enhance urban resilience by supporting seamless cross-regional EM information transfer (15), ensuring patients receive continuous care regardless of their location within the event’s scope. Its characteristics of “peacetime-wartime combination” and dual function in both event support and disaster relief highlights its strategic value. During the events, they can be rapidly deployed to the venue, and in the event of a disaster, they can be immediately redirected to the affected area. This dual-use infrastructure model significantly reduces equipment idleness and optimises long-term operational and maintenance costs.

### Breakthrough in EM innovation

Despite differences in health characteristics between event participants and nursing home residents, the underlying principles of healthcare delivery—namely, the effectiveness of on-site treatment, the necessity of referral, and the continuity of care—are highly similar. The application of MH in sporting events is essentially a contextual extension of the “Hospital at Hand” concept (19). MH not only address the fluctuating demand for medical resources during events but also provide reusable infrastructure for EM systems. This innovative EM approach not only redefines the paradigm of medical support for large-scale events but also offers a robust solution for building resilient healthcare systems. Its strategic value extends beyond event medical care, becoming a crucial pillar for the modernisation of public health governance.

## Practices of MH in EM supports for large-scale sports events

### 2024 Tour of Shanghai New Town Cycling Race

The Tour of Shanghai New City Cycling Race, held in September 2024, spanned approximately 300 kilometres across five administrative districts: Jiading, Qingpu, Songjiang, Fengxian, and Nanhui (20). During the event, the MH of Huashan Hospital deployed a command vehicle, outpatient vehicles, an X-ray vehicle, a surgical vehicle, a power supply vehicle, and an ambulance with monitoring capabilities. A professional medical team accompanied the whole race and were deployed at key points along the route to ensure the smooth progression of the competition. The event was challenged by typhoon weather, which led to slippery roads and a significant increase in accident risk. Under these conditions, the MH treated dozens of injured individuals, with only five requiring transfer to designated hospitals for further treatment (21).

### 2024 Shanghai Marathon

The 2024 Shanghai Marathon, held on 1 December, attracted approximately 38,000 runners (22). At the finish line area, the MH of Huashan Hospital deployed a command vehicle, an X-ray vehicle, a surgical vehicle, and medical team. For common runner injuries such as muscle strains, abrasions, dehydration, and fractures, the MH provided on-site treatment to nearly a hundred patients, with only two requiring transfer to designated hospitals.

The practical application of MH in both the Tour of Shanghai New City Cycling Race and the Shanghai Marathon verified its advantages of rapid deployment and on-site first aid. Under the complex geographical environments and high-density event scenarios, MH established a EM support network, enabling precise allocation and efficient utilisation of EM resources. This demonstrates a replicable and scalable solution for large-scale event EM support.

## Challenges and development

Despite the significant advantages demonstrated by MH in providing EM support for sports events, its practical application continues to face multiple challenges and requires systematic innovation to drive future development.

### Logistical complexity and deployment site adaptability

Although MH is self-sufficient, the diversity of deployment scenarios—such as remote mountainous regions or dense urban centres—imposes stringent demands on logistical support. Prior to deployment, it is crucial to precisely identify flat, open sites and ensure a continuous supply of water and electricity. At the same time, robust plans for medical waste disposal and the transportation of heavy equipment must be established to suit varying environmental conditions. Furthermore, the entry and exit of large medical vehicles in traffic-dense areas can lead to congestion or safety hazards (15). This necessitates collaborative development of dynamic traffic management strategies with event organisers and traffic authorities to balance event security and public safety requirements.

### Personnel training, institutional collaboration, and multidisciplinary integration

The efficient operation of MH relies on versatile personnel and inter-agency collaboration. Operating staff must not only be proficient in medical techniques but also familiar with the specialised equipment inherent to mobile units, including portable imaging devices and emergency communication systems. Experience from the Chongqing MH indicates that insufficient operational training directly compromises rescue efficiency (15). Moreover, it is essential to establish standardised collaboration protocols with local hospitals, emergency services (police, fire), and event organisers. This is vital to prevent conflicts of responsibility between city-level emergencies (e.g., natural disasters) and event-specific medical plans, ensuring a clear chain of command. Future development should focus on integrating multidisciplinary teams, including sports medicine specialists, emergency physicians, nursing staff, and logistics engineers, to form a cohesive “medical-technical-management” response system. This integrated approach aims to optimise the entire chain, from casualty identification to resource allocation.

### Opportunities for technology integration and innovation

Technological advancements for MH must prioritise lightweight design, intelligent systems, and comprehensive integration. Future development should focus on more portable and rapidly deployable medical units, incorporating automated setup procedures (e.g., one-button tent deployment) to significantly reduce response times. Simultaneously, establishing real-time data collection and analysis systems is crucial for intelligent management of patient tracking, resource allocation, and risk early warning, thereby enhancing operational efficiency (5, 6). Epidemiological research specifically targeting sports-related injuries, such as cranial trauma in cycling races or heatstroke in marathons, can provide precise data to inform and refine future event medical support strategies. Furthermore, the evolution of MH should extend beyond individual medical units to encompass a holistic system design that integrates logistics, communication, and event management, ensuring seamless connectivity across all operational phases. By regularly participating in urban emergency drills and community medical services, MH can transition into routine medical resources. This approach addresses the challenge of traditional emergency assets being “heavy on acquisition but light on maintenance”, fostering sustainable “peacetime-wartime integration”.

## Conclusion

MH is a strategic, highly mobile solution to provide comprehensive healthcare coverage for sporting events. Their proven ability to provide further care on the spot, effectively avoid unnecessary transport to traditional eds, and facilitate seamless continuity of care has significantly reduced the burden on urban emergency medical services. In addition, mobile hospitals have demonstrated unique and unrivaled advantages in complex cross-administrative, multi-stage competitions, providing dynamic, adaptive, and customized medical coverage that cannot be provided by fixed medical infrastructure.

## Data Availability

Publicly available datasets were analyzed in this study. This data can be found here: https://www.citynewsservice.cn/service/ICYMI-Special-Edition-Get-Ready-to-Pedal-in-the-Tour-of-Shanghai-2024-%E2%80%93-And-We’ve-Got-FREE-Spots-4kbyrgnx.
